# *Listeria monocytogenes* as a Vector for Cancer Immunotherapy: Current Understanding and Progress

**DOI:** 10.3390/vaccines6030048

**Published:** 2018-07-25

**Authors:** John C. Flickinger, Ulrich Rodeck, Adam E. Snook

**Affiliations:** 1 Department of Pharmacology and Experimental Therapeutics, Thomas Jefferson University, 1020 Locust Street, Philadelphia, PA 19107, USA; John.Flickinger@jefferson.edu; 2 Department of Dermatology, Thomas Jefferson University, 1020 Locust Street, Philadelphia, PA 19107, USA; Ulrich.Rodeck@jefferson.edu

**Keywords:** *Listeria*, cancer, vaccine, immunotherapy, bacteria

## Abstract

*Listeria monocytogenes*, a Gram-positive facultative anaerobic bacterium, is becoming a popular vector for cancer immunotherapy. Indeed, multiple vaccines have been developed utilizing modified *Listeria* as a tool for generating immune responses against a variety of cancers. Moreover, over a dozen clinical trials testing *Listeria* cancer vaccines are currently underway, which will help to understand the utility of *Listeria* vaccines in cancer immunotherapy. This review aims to summarize current views on how *Listeria*-based vaccines induce potent antitumor immunity and the current state of *Listeria*-based cancer vaccines in clinical trials.

## 1. Introduction

The proficiency of the immune system to recognize and eliminate cancer cells, a process summarily called immunosurveillance, is well documented [1]. Failure of immunosurveillance enables the clinical development of cancer and has motivated the search for strategies to rearm and restore effective immune responses to malignant cells. One such strategy includes vaccine development against cancer. In general, cancer vaccines are composed of antigens found in tumor cells, often referred to as tumor-associated antigens (TAA), paired with adjuvants designed to induce an immune response. Antigen-specific T-cell responses induced by cancer vaccines have the potential to produce more targeted elimination of cancer cells than conventional chemotherapy, as well as lead to durable memory responses capable of challenging cancer recurrence. This review aims to summarize one particular approach to cancer vaccination, employing *Listeria monocytogenes* vectors, and its current progress in terms of preclinical development and clinical trials.

*Listeria monocytogenes* (Lm) is a Gram-positive bacteria most widely known for its ability to infect humans and produce a variety of symptoms, including gastroenteritis, meningitis, and encephalitis [2]. In general, the human immune system mounts potent innate and adaptive immune responses capable of controlling Lm infections. As a result, serious infection by Lm is rare and typically limited to elderly, pregnant, or immunocompromised patients [2]. Decades worth of research on the properties that make Lm immunogenic and how these properties can be exploited have led to its advancement as a vaccine platform for cancer immunotherapy in clinical trials.

Lm has numerous features that make it an attractive vector for cancer immunotherapy. While other vectors may be inhibited through neutralizing antibodies, Lm infection triggers only modest humoral responses which fail to block reinfection [3,4]. This permits repeated administration of Lm-based vectors to boost T-cell responses in patients as needed. Additionally, and in contrast to DNA or peptide vaccines, Lm strongly induces both innate and adaptive immune responses. Indeed, the ability of bacteria to activate innate immune mechanisms and elicit antitumor responses was first appreciated over 100 years ago when William Coley observed instances of spontaneous cancer regression in sarcoma patients who acquired bacterial skin infections [5]. Beyond innate immune mechanisms, and in contrast to other bacterial vectors, such as *Salmonella*, Lm is a potent stimulator of cytotoxic lymphocytes and cell-mediated immunity [4]. Over 50 years ago, George Mackaness further demonstrated that mice exposed to sublethal doses of Lm developed long-lived, antibody-independent immune responses which protected against a future Lm challenge administered at lethal doses [6]. These and other observations eventually led to the exploration of Lm as a vaccine vector with the aim of inducing similar cell-mediated immune responses towards foreign antigens. This concept was reduced to practice by Paterson and colleagues using Lm-expressing β-galactosidase antigen to induce a cytotoxic lymphocyte response that killed cancer cells expressing β-galactosidase [7]. Since the publication of this seminal paper over 25 years ago, numerous Lm vaccines have been developed for the treatment of a variety of cancers. Here, we will provide an overview of the mechanisms underlying Lm-based cancer vaccines and summarize the current state of Lm vaccines in cancer clinical trials.

## 2. Pathogenesis of *Listeria* Infection

**Figure 1:**
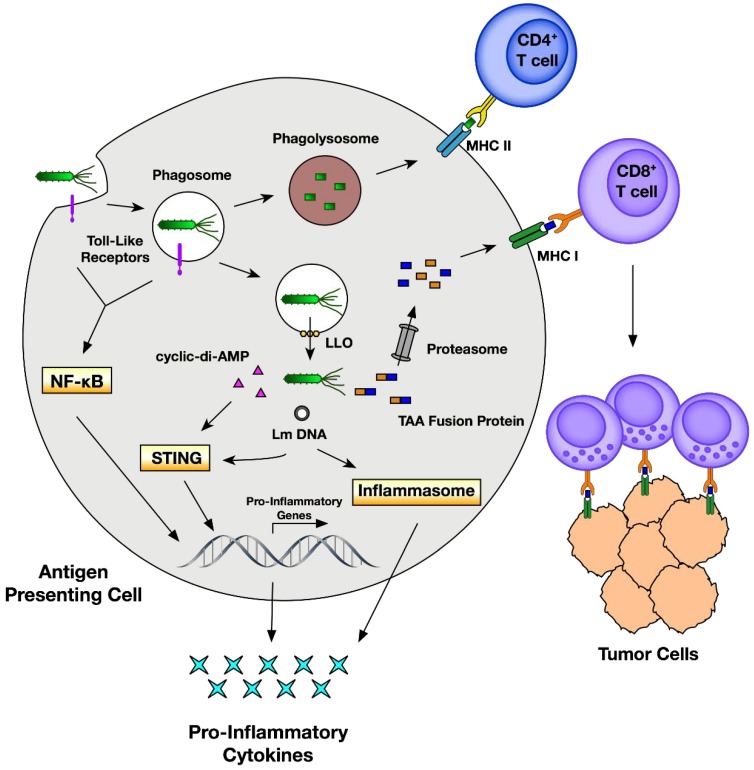
Innate and adaptive immune responses to recombinant *Listeria*. *Listeria monocytogenes* (Lm) is internalized by antigen-presenting cells into phagosomes. During entry, Lm is sensed by toll-like receptors leading to the activation of NFκ-B and synthesis of pro-inflammatory genes. Phagosomes may then fuse with lysosomes to form phagolysosomes where Lm can be killed, leading to loading of its antigens onto MHC class II for activation of CD4^+^ helper T cells. Alternatively, Lm can express the pore-forming toxin listeriolysin O (LLO) to perforate phagosomes and gain entry into the cytosol. Once in the cytosol, recombinant Lm secretes tumor-associated antigens (TAA) as fusion proteins with Lm antigens into the cytosol where they can be degraded by proteasomes and loaded onto MHC class I for activation of TAA-specific CD8^+^ cytotoxic T lymphocytes. Additionally, cytosolic Lm triggers further induction of pro-inflammatory pathways through secretion of the cyclic dinucleotide cyclic-di-AMP and detection of Lm DNA. Secreted cyclic-di-AMP directly stimulates the STING pathway and negatively regulates the NF-κB inhibitor RECON (not shown) [18], while the presence of genomic Lm DNA can lead to the activation of STING and inflammasome pathways, both of which contribute to the transcription of pro-inflammatory genes and cytokines.

In nature, Lm typically enters through the gastrointestinal system after ingestion of contaminated food, where it crosses the intestinal epithelium and spreads into the bloodstream [2]. However, in the context of immunotherapeutic applications, Lm is largely administered intravenously, bypassing the intestinal epithelium. Once in the bloodstream, Lm can enter a variety of organs, including the liver, brain, and placenta [2,8]. While it can survive in the extracellular environment, its preferred niche is the cell cytoplasm where it undergoes replication [9]. As Lm transitions into intracellular compartments, numerous immune mechanisms are activated. These mechanisms are essential for understanding Lm vaccine therapy and are illustrated in Figure 1.

Lm entry into mammalian cells is primarily mediated through a family of proteins called internalins. Of particular interest are the internalin A (InlA) and internalin B (InlB) proteins, which guide Lm invasion through binding to receptors on host cells and triggering receptor-mediated endocytosis [8]. Importantly, InlA and InlB bind to different receptors and promote infection of different cell types. InlA binds E-cadherin which is found on epithelial cells, while InlB interacts with the hepatocyte growth factor, Met [10,11]. In addition to entry via cell surface receptors, Lm is actively taken up via phagocytosis by antigen-presenting cells (APCs) [2]. Importantly, as Lm interacts with the outside surfaces of mammalian cells, innate immune responses against Lm begin to mount. On the surface of the cell, as well as inside phagosomes, pathogen-associated molecular patterns (PAMPs) expressed by Lm are recognized by mammalian Toll-like receptors (TLRs), leading to the activation of NF-κB signaling and the expression of pro-inflammatory genes [2,12].

Upon internalization into phagosomes, it appears that Lm can undergo one of two fates. It is estimated that the majority of bacteria are killed upon phagosome–lysosome fusion [13], providing a source of antigens for MHC class II-dependent presentation and priming of Lm-specific CD4^+^ T-cell responses [4]. However, Lm has evolved mechanisms to escape lysosomal degradation and enter the cytosols of infected cells by a well-defined mechanism. As Lm transitions from the extracellular environment into the host cell, it begins to express the transcription factor PrfA [9]. PrfA activates numerous virulence genes, including those responsible for phagosome escape, such as the pore-forming toxin listeriolysin O (LLO) and two different phospholipases, PlcA and PlcB. Secreted LLO molecules oligomerize at the phagosomal membrane to form a β-barrel pore-like structure through which Lm can pass [14], while phospholipases mediate exit through direct hydrolysis of membrane lipids [15]. Once outside the phagosome, Lm-secreted peptides enter the host cell cytosol where they can be degraded by proteasomes and loaded onto MHC class I molecules for presentation to cytotoxic T cells [4]. The direct secretion of antigens into the cytosol, as well as prior degradation in phagosomes, results in the induction of potent CD4^+^ and CD8^+^ T-cell responses to Lm antigens [4,16,17].

Having escaped the phagosome, Lm is free to move throughout the host cell cytosol via expression of the virulence factor actin assembly-inducing protein (ActA). Anchored to the surface of Lm, ActA protein interacts with the Arp2/3 complex to promote nucleation and polymerization of actin monomers into filaments [19]. Forces generated via actin polymerization are capable of projecting Lm throughout the cytosol as well as into the plasma membrane of the infected cell, forming a protrusion that can be internalized by a neighboring cell and leading to dissemination of the infection. [19].

Similar to the sensing of Lm on the cell surface and inside phagosomes by toll-like receptors, additional pattern recognition receptors recognize Lm in the cytosol and further stimulate pro-inflammatory pathways. One route is through the activation of retinoic acid-inducible gene I (RIG-I)-like receptors by Lm RNA, leading to the activation of the mitochondrial antiviral signaling protein (MAVS) and transcription of interferon (IFN) genes [20,21]. Further transcription of IFN genes is mediated through activation of the STING pathway through at least two mechanisms. First, Lm can secrete cyclic diadenosine monophosphate (cyclic-di-AMP), directly activating STING [22]. Second, Lm lysis can expose genomic DNA leading to activation of the cyclic GMP-AMP synthase, production of cGAMP, and activation of STING [23]. Regardless of the route, activation of the STING pathway promotes the transcription of type I IFN genes, including IFN-β [22,24]. Importantly, IFN-β and activation of the STING pathway improve the priming of CD8^+^ T cells in the tumor microenvironment, leading to potent antitumor responses [25,26]. Paradoxically, Lm mutants, secreting higher levels of cyclic-di-AMP, elicit reduced T-cell responses, suggesting that the role of STING activation in Lm infection is not fully understood [24]. Finally, Lm infection can activate NLRP3 and AIM2 receptors, leading to activation of the inflammasome and further increasing pro-inflammatory cytokines [2,27].

## 3. Development of Attenuated *Listeria* Strains for Vaccination

Because wild-type *Listeria* is pathogenic and not suitable for clinical use, considerable effort has been expended to improve Lm safety. The ideal Lm strain would minimize pathogenicity while maximizing immunogenicity towards the target antigen. Multiple strategies to attenuate Lm have been developed towards that end.

### 3.1. Deletion of Virulence Genes

A widely employed strategy to attenuate Lm involves deleting genes responsible for Lm tropism and cell-to-cell transmission. As mentioned previously, Lm tropism into non-phagocytic cells, such as hepatocytes, is mediated by the virulence factor internalin B. Therefore, deletion of *inlB* is expected to limit liver toxicity and render phagocytic antigen-presenting cells the primary target of Lm infection. Indeed, Δ*inlB* mutants display reduced hepatocyte entry while infecting monocytes as effectively as wild-type strains [28]. Further attenuation can be achieved by deleting the virulence factor *actA* [28]. In wild-type Lm, ActA expression allows Lm to polymerize host actin filaments to maneuver through the host cytosol and spread from one cell into another [2,9]. Deletion of *actA* is thus expected to confine Lm to the cytoplasm of the cell it initially infects, limiting the spreading of infection and reducing toxicity. Evidence for this is supported by experiments demonstrating that Δ*actA* strains have a much higher LD_50_ (>1000-fold) than wild-type strains [29]. By deleting both *inlB* and *actA*, it is possible to selectively infect antigen-presenting cells and reduce off-target toxicity. Vaccines using Δ*actA*/Δ*inlB* strains also exhibit rapid clearance of infection from the liver and spleen compared to single Δ*actA* or Δ*inlB* mutants [28]. Importantly, despite a rapid clearance of Lm, Δ*actA*/Δ*inlB* strains demonstrate comparable antitumor responses [28]. The Lm Δ*actA*/Δ*inlB* strain, also known as Live Attenuated Double-Deleted (LADD) (Aduro BioTech Inc., Berkeley, CA, USA), forms the basis for many vaccines in clinical trials.

### 3.2. Episomal Replacement of Virulence or Metabolic Genes

While attenuation can be achieved through gene deletion, the deletion of a gene followed by reintroduction of that gene via plasmid can also result in attenuation. It is likely that episomal gene expression leads to different quantities or spatial patterns of gene product than expression from the host genome [17]. As described above, PrfA is a master transcription factor that regulates the transcription of multiple Lm virulence factors, including LLO, ActA, and phospholipases [9]. Indeed, Lm strains deficient in *prfA*, such as XFL-7, are severely attenuated [30,31]. Moreover, due to the lack of LLO and phospholipase expression, *prfA* mutants are incapable of phagolysosome escape and invasion into the host cytosol [31]. Partial restoration of virulence is achieved by the addition of plasmid-encoded PrfA into which the tumor-associated antigens (TAA) are cloned as well [30,32,33]. The result is an immunogenic strain that is significantly attenuated compared to wild-type Lm [30,32,33].

A similar attenuation strategy has been employed utilizing the Δ*dal*/Δ*dat* strain (Lmdd). Lm requires the *dal* and *dat* genes to synthesize D-alanine to build peptidoglycan and lipoteichoic acid [34]. Strains that are deficient in both of these genes fail to replicate in vitro unless supplemented with exogenous alanine [35,36]. Furthermore, immunogenicity of the Lmdd strain in vivo depends on co-administration of alanine [37]. However, by transforming the Lmdd strain with a plasmid encoding the dal gene under a constitutively active promoter, it is possible to partially restore virulence and achieve immunogenicity without alanine supplementation [38]. Often, the Lmdd strain is further attenuated by the deletion of *actA* creating what is often called the LmddA strain [38,39]. Both the LmddA and the XFL-7 strain are currently being evaluated in clinical trials by Advaxis Inc., Princeton, NJ, USA.

### 3.3. Killed but Metabolically Active

Heat-killed Lm and strains deficient in phagolysosomal escape have long been noted to be poor inducers of CD8^+^ T-cell responses [40,41], suggesting that Lm entry into the host cytosol is required for the effective activation of CD8^+^ T cells. As a result, Lm vaccine development has relied on attenuated, rather than killed, Lm formulations. However, live attenuated Lm strains are capable of inducing listeriosis in some cases and may not be safe to use in immunocompromised patients [4,42]. In an effort to further improve the safety profile of Lm vaccines, killed but metabolically active (KBMA), Lm vaccines have been developed. Lm strains deficient in nucleotide excision repair can be photochemically inactivated by the DNA crosslinking agent psoralen coupled with UV irradiation [43]. This process induces irreversible DNA damage and renders Lm incapable of replication. However, for a period of hours after photochemical treatment, Lm is capable of invading host cells, escaping into the cytosol, and secreting TAA products to elicit antitumor CD8^+^ T-cell responses [43,44]. Initially, KMBA vaccines were found to invoke inferior protective responses compared to live, attenuated vaccines [45]. However, enhanced CD8^+^ T-cell responses have been achieved using KBMA vaccines with a constitutively activating *prfA* mutation [44]. While promising, KBMA Lm vaccines have yet to enter clinical trials.

## 4. Fusion with *Listeria* Antigens Enhances Antitumor Responses

While it is possible to generate therapeutic immune responses by engineering Lm to express a TAA alone, nearly all Lm vaccines express TAAs as chimeric proteins fused to a native Lm antigen. Initial studies piloting Lm vaccines fused TAAs to highly-secreted Lm proteins as a method to increase the delivery of TAAs into the cytosols of host cells [46]. However, it is now well appreciated that certain Lm proteins, when fused to TAA, can also act as adjuvants, boosting therapeutic efficacy. The two most common fusion partners with TAAs include modified versions of LLO and ActA.

### 4.1. Listeriolysin O

Listeriolysin O (LLO) is a virulence factor in the Lm life cycle and perforates the phagosome to aid Lm entry into the cytosol. Because LLO could also perforate the plasma membrane, vaccines incorporating LLO fusion proteins are typically engineered to express it as a truncated version (tLLO) which is deficient in the hemolytic domains necessary for pore formation [30,33]. Effective antitumor immunity using tLLO-TAA constructs was first demonstrated by Gunn et al. [30]. In these studies, mice were injected with a cancer cell line expressing the human papillomavirus (HPV) antigen E7 and then vaccinated with Lm expressing E7 alone or E7 as a fusion protein with tLLO (tLLO-E7). While E7 expressing Lm had almost no effect on tumor growth, immunization with tLLO-E7-expressing Lm induced complete regression of 75% of the tumors [30]. Since these initial observations, numerous studies examining the role of tLLO in antitumor immunity have been conducted, and it is believed that tLLO enhances vaccine responses through a combination of different mechanisms.

It has been suggested that tLLO enhances the delivery of TAA to the proteasome, facilitating antigen processing and MHC class I presentation [47]. Indeed, LLO contains PEST-like sequences and destabilizing N-terminal residues that enhance protein degradation and affect cytosolic LLO levels [48,49,50]. This hypothesis has been supported by direct comparison of Lm vaccines expressing either E7 alone, E7 fused to the isolated PEST sequence (PEST-E7), E7 fused to a version of tLLO lacking the PEST sequence (ΔPEST-E7), or the full tLLO sequence fused to E7 (tLLO-E7). Immunization with PEST-E7 elicited increased CD8^+^ T-cell responses and antitumor immunity compared to vaccination with E7 alone [47], suggesting that PEST sequences can improve vaccine responses towards TAAs. Consistent with this view, vaccination with ΔPEST-E7 generated reduced CD8^+^ T-cell responses and antitumor immunity compared to tLLO-E7 [47]. However, immunization with ΔPEST-E7 also elicited superior antitumor responses compared to the E7 vaccine, suggesting that tLLO may enhance responses through additional mechanisms independent of PEST sequences [32,47]. One possibility is that tLLO may signal through pattern recognition receptors. Indeed, LLO and other pore-forming toxins can activate TLR4 [51]. Initially, these immunogenic properties were thought to depend on LLO cholesterol-binding domains which are involved in LLO-induced pore formation [51]. However, later experiments indicated that even non-hemolytic forms of LLO are capable of inducing dendritic cell maturation and the synthesis of proinflammatory cytokines, suggesting that LLO might be signaling through pattern recognition receptors independent of its cytotoxic attributes [52]. However, this study was unable to identify a specific pattern recognition receptor responsible for these effects.

Since many TAAs targeted by vaccines are also expressed by normal tissues, effective vaccines must overcome tolerance [4]. Importantly, multiple lines of evidence suggest that tLLO-TAA fusion constructs can remodel the immunosuppressive tumor microenvironment and overcome tolerance mechanisms. While Lm expressing E7 alone elicits poor antitumor responses, its effectiveness is substantially increased by regulatory T-cell depletion [30]. Moreover, experiments examining tumor-infiltrating CD4^+^CD25^+^ regulatory T cells (Tregs) revealed that vaccination with Lm expressing E7 alone increased the abundance of Tregs, while the tLLO-E7 vaccination decreased Tregs [53,54], supporting the hypothesis that tLLO may enhance antitumor responses through changes in Tregs. These results may be partly explained by a preferential expansion of CD4^+^Foxp3^−^ cells upon vaccination with tLLO-E7 [54]. Interestingly, vaccination with Lm strains expressing tLLO alone appears to affect the CD4^+^ effector and regulatory T-cell numbers similarly to those obtained with the tLLO-E7 fusion protein [54]. In addition, tLLO-TAA constructs reportedly reduce the number and functional activity of myeloid-derived suppressor cells (MDSCs) in the tumor microenvironment, potentially contributing to the ability of Lm to break peripheral tolerance against TAAs [55]. Interestingly, immunity induced by tLLO–TAA fusion constructs does not appear to be limited by central tolerance mechanisms. Experiments using transgenic mice expressing the E7 antigen in the thymus, resulting in central tolerance to E7, demonstrated that tLLO-E7 vaccines could expand low-avidity E7-specific T cells and produce antitumor responses [56]. Additionally, tLLO fusions widen the breadth of T-cell receptors (TCRs) recognizing self-antigen. In support of this notion, DNA vaccines containing a fusion of the self/tumor antigen HER2 with tLLO induced T-cell responses towards subdominant epitopes [32]. In this study, DNA vaccines encoding HER2 elicited responses to only two HER2 epitopes, while a tLLO-HER2 vaccine elicited responses against four HER2 epitopes [32]. However, Lm-based, rather than DNA-based, vaccines expressing HER2 fragments fused with tLLO induced response against nine HER2 epitopes, suggesting that even broader responses are generated in the context of Lm [32].

### 4.2. ActA

A different widely used strategy for Lm vaccination involves fusing TAAs to ActA. Similar to fusion with tLLO, these approaches tend to rely on truncated ActA sequences of varying lengths. Some investigators have used the first 100 amino acids of ActA [44,57], while others used the first 420 amino acids of the protein [58,59]. Similar to tLLO, fusion of E7 with ActA produces superior antitumor immunity compared to E7 alone [60]. However, explanations for superior therapeutic responses are incomplete. Like tLLO, ActA contains N-terminal PEST domains which may facilitate proteasomal degradation and enhance TAA processing [4,61]. However, experiments have also suggested that ActA may exert immunostimulatory effects independent of ActA–TAA fusion. Using protein vaccines containing either ActA, E7, or ActA covalently conjugated to E7 (ActA-E7), immunization with a mixture of ActA and E7 produced similar CD8^+^ T-cell responses and antitumor immunity compared to vaccination with ActA-E7 fusion protein [60]. It has been speculated that ActA may possess PAMP-like attributes, leading to the production of pro-inflammatory cytokines and maturation of antigen-presenting cells [60]. Much like tLLO, the precise mechanisms for its adjuvant-like properties are not entirely understood.

At least two studies have directly compared Lm vaccines expressing ActA-E7 to those expressing tLLO-E7 [56,59]. Vaccination with either ActA-E7 or tLLO-E7 resulted in similar levels of circulating E7-specific CD8^+^ T cells and yielded similar antitumor effects [59]. Interestingly, vaccination with tLLO-E7 produced higher percentages of tumor-infiltrating E7-specific CD8^+^ T cells [58,59]. Both vaccines generated similar levels of E7-specific responses in transgenic mice expressing E7, indicative of a comparable potency in breaking tolerance [56]. While it is unclear which strategy, if any, is superior, it is well-established that fusion of TAA to either ActA or tLLO can boost immune responses towards TAAs and enhance antitumor immunity [4,30].

## 5. *Listeria* Vaccines in Clinical Trials

To date, over 30 clinical trials testing 10 different Lm cancer vaccines have been initiated (Table 1). Two companies have been at the forefront of advancing Lm-based vaccines through clinical trials, with each using unique approaches. In general, Lm-based vaccines developed by Aduro BioTech Inc. are derived from the LADD^®^ strain. TAA genes are cloned into integration vectors allowing for stable incorporation into the Lm genome [62]. Expression of TAAs is regulated by the ActA promoter and occurs as a fusion protein with a modified form of ActA [17]. In contrast, Lm vaccines manufactured by Advaxis Inc. are based on either the XFL-7 or the LmddA strains described above. Expression of TAAs occurs episomally as a fusion protein with tLLO under control of the hly (LLO) promoter [38,39].

### 5.1. ADXS11-001 (Axalimogene Filolisbac [AXAL])

ADXS11-001, also known as Axalimogene Filolisbac or AXAL, is a vaccine against cancers caused by the human papillomavirus (HPV). HPV expresses the E6 and E7 oncoproteins which directly promote cell division, genomic instability, and tumorigenesis by interfering with the functions of the tumor suppressor proteins p53 and RB [63]. Approximately 5% of all cancers world-wide are caused by HPV infection, with most cases being attributed to HPV subtypes 16 and 18 [64]. AXAL is based on the XFL-7 strain, engineered to secrete the E7 protein from HPV 16 fused to tLLO [30,58,59]. Numerous preclinical studies have demonstrated its ability to induce regression of HPV-transformed tumors, leading to its advancement to multiple clinical trials [30,58,59].

Most clinical trials studying AXAL have been in patients with cervical cancer. After a Phase I clinical trial demonstrated safety [65], multiple Phase II trials were initiated. Results from a Phase II trial in India comparing AXAL and AXAL plus cisplatin to a historical control were recently published [66]. In this trial, patients with refractory cervical cancer received either three doses of AXAL or four doses of AXAL with five infusions of cisplatin. While median overall survival (OS) was equivalent, patients receiving AXAL or AXAL plus cisplatin experienced 1.5 to 2-fold increases in overall survival at 12 months (34.9%) and 18 months (24.8%) [66]. In addition to combination with chemotherapy, AXAL is also being tested in combination with immune checkpoint inhibitors. Preclinical models using AXAL with αPD-1 inhibitors have demonstrated a reduction in Treg and MDSC abundance and an increased antitumor response compared to AXAL alone [75]. A Phase I/II trial combining AXAL with the αPD-1 inhibitor durvalumab was initiated but briefly put on hold after a patient died of respiratory failure (ClinicalTrials.gov identifier NCT02291055). After an investigation, the FDA reinstated this trial and it is currently ongoing. Of great interest, AXAL was recently advanced into the double-blind, randomized Phase III AIM2CERV clinical trial, comparing AXAL to a placebo in patients with high-risk, locally-advanced cervical cancer (ClinicalTrias.gov identifier NCT02853604). This is currently the only Phase III trial testing an Lm-based vaccine. Patients in the AIM2CERV trial will receive chemoradiation with curative intent followed by multiple infusions of AXAL or placebo. Dosing of AXAL or the placebo is to occur every 3 weeks for the first 3 months, followed by an infusion every 8 weeks for a total of five doses or until disease recurrence. The primary endpoint will compare disease-free survival (DFS) in patients receiving AXAL or placebo and is expected to be met in June 2020.

While most trials with AXAL have been in cervical cancer, AXAL is also being pursued in patients with HPV-associated oropharyngeal, anal, and lung cancers. In oropharyngeal cancer, a Phase I trial was terminated early after a patient suffered a dose-limiting toxicity (ClinicalTrials.gov identifier NCT01598792). Despite this setback, a Phase II trial testing AXAL as a monotherapy in anal cancer continues to recruit patients (ClinicalTrials.gov identifier NCT02002182). In anal cancer, a Phase I trial studying AXAL in combination with radiation and the chemotherapies mitomycin and 5-FU was recently completed (ClinicalTrials.gov identifier NCT01671488). Of the nine patients who completed treatments, eight were progression-free at a median follow-up time of 42 months [67]. Based on those results, a subsequent Phase II trial assessing AXAL as a monotherapy in anal cancer was initiated (ClinicalTrials.gov identifier NCT2399813). This trial is expected to be completed in March 2022.

In addition to AXAL, Advaxis is also pursuing a second-generation version called ADXS-DUAL in collaboration with Bristol-Myers Squibb. ADXS-DUAL possess E7 from both HPV 16 and HPV 18 serotypes. Although the majority of clinical trials by Advaxis have focused on AXAL, Advaxis is also advancing Lm vaccines against the tumor antigens PSA (prostate-specific antigen) and HER2 (human epidermal growth factor 2).

### 5.2. ADXS31-142

PSA is a serine protease whose expression is normally confined to the prostate. While its utility as a serum diagnostic marker in prostate cancer remains controversial, it is well-accepted that PSA can act as an immunotherapeutic target in prostate cancer [76]. Early studies piloting tLLO-PSA vaccines were based on the XFL-7 strain of Lm; however, clinical studies use ADXS31-142 based on the LmddA strain. Both versions have been shown to modestly reduce tumor burden and the number of tumor-infiltrating Tregs in mice inoculated with the prostate cancer cell line TPSA23 [33,38]. Based on the hypothesis that radiation therapy may induce immunogenic tumor cell death and promote favorable inflammatory environments for T-cell activation, follow-up studies assessed ADXS31-142 in combination with radiation therapy [77,78]. While ADXS31-142 or radiation alone slowed TPSA23 tumor growth, combination therapy led to multiple instances of complete tumor regression [77]. Synergistic antitumor responses were associated with significant increases in PSA-specific T cells in the spleen and tumor [77] as well as enhanced intratumoral Th1 responses [78]. Recent reports have demonstrated an additional therapeutic benefit by combining ADXS31-142 and radiation therapy with αPD-1 or αPD-L1 inhibitors [78].

Currently, ADXS31-142 is being tested in a Phase I/II trial in patients with metastatic castration-resistant prostate cancer. The trial (ClinicalTrials.gov identifier NCT02325557), also known as the KEYNOTE-046 trial, is assessing progression-free survival following ADXS31-142 alone or in combination with the αPD-1 antibody pembrolizumab. While the trial is not yet completed, an early report found that patients vaccinated with ADXS31-142 generated T-cell responses against multiple prostate antigens beyond PSA through a process known as epitope spreading [68]. In this context, it is believed that PSA-specific T cells inducing prostate cancer cell death and the production of inflammatory cytokines result in the release of additional tumor antigens and induction of immune responses against additional epitopes, ultimately broadening the therapeutic scope of the vaccine.

### 5.3. ADXS31-164

HER2 is a receptor tyrosine kinase that is frequently overexpressed by cancers, including breast, salivary gland, and bone [79,80]. HER2 expression is often associated with poor prognosis, as HER2 signaling has been shown to directly activate pathways responsible for tumor growth and survival [79]. While antibody-based therapies targeting HER2 have been developed, many patients still experience recurrence after treatment. In addition to antigen escape, antibody-based therapies against HER2 primarily induce antibody-dependent cytotoxicity in the absence of adaptive immune responses. Indeed, poor Th1 responses after anti-HER2 antibody therapy strongly correlated with recurrence, leading to speculation that vaccines boosting Th1 responses could improve the standard of care [81,82].

Due to its large molecular mass, early Lm-based HER2 vaccine designs consisted of five different Lm vaccines, with each expressing tLLO fused to a unique region of the rat HER2 extracellular or intracellular domain [32]. Individually, each Lm strain antagonized the growth of the HER2 expressing NT-2 tumor cell line to a similar extent. Based on this proof of concept, a single Lm vaccine against human HER2, known as ADXS31-164, was subsequently developed [39,83]. ADXS31-164 is derived from the attenuated LmddA strain and is engineered to secrete a chimeric tLLO fusion protein containing immunogenic regions from two extracellular domain sequences and one intracellular domain sequence of HER2 [83]. The chimeric HER2 protein is designed to encompass most of the known human MHC class I epitopes of the HER2 protein. Preclinical studies with this vaccine demonstrated the ability of ADXS31-164 to reduce the number of regulatory T cells in tumors and increase the CD8^+^ T-cell/Treg ratio [39]. Impressive results with ADXS31-164 in preclinical studies has led to its advancement to clinical trials in canines and humans.

Recently, results from a Phase I trial of ADXS31-164 in canine osteosarcoma were published [84]. In this trial, canines received three doses of ADXS31-64 following amputation and chemotherapy. Out of 18 immunized dogs, 15 developed T-cell responses against HER2. Impressively, this led to a significant reduction in metastatic disease and increase in survival. Canines vaccinated with ADXS31-164 had a median survival time of 956 days, and 56% were still alive three years after chemotherapy, compared to a historical control of 423 days and 22%, respectively [84]. Canines with immune responses to the intracellular HER2 domain had the most robust effects on survival, potentially related to the fact that the intracellular HER2 domain is indispensable for oncogenic cell signaling [85]. In contrast to HER2/neu antibodies, which are encumbered by adverse effects, including cardiotoxicity [86], ADXS31-164 was not associated with cardiotoxicity in canines [84]. In 2014, Advaxis licensed the use of ADXS31-164 in canines to Aratana Therapeutics Inc. where it was renamed AT-014. The USDA, in early 2018, granted conditional clinical approval for the use of AT-014 in canine osteocarcoma. Currently, AT-014 is the only Lm vaccine to receive any clinical approval. While data from mice and dogs indicate promising potential, results from human trials have yet to be released. Currently, an ongoing Phase I/II trial is examining the use of ADXS31-164 in HER2 expressing solid tumors. This trial is expected to be completed in December of 2018.

### 5.4. CRS-100 (ANZ-100)

While most antitumor effects of Lm vaccines are attributed to Lm secretion of TAAs leading to TAA-specific T-cell responses, studies utilizing CRS-100 highlight the powerful effects that Lm vaccines can have on the innate immune system. CRS-100 consists of the live, attenuated Δ*actA*/Δ*inlB* strain of Lm and does not express exogenous antigens. Despite this, the CRS-100 vaccination has been shown to mediate antitumor effects, primarily through inducing antigen-independent NK cell activity. In preclinical modes of mice with hepatic colorectal cancer metastases, the CRS-100 vaccination induced recruitment of NK cells to the liver, leading to antitumor responses [87]. The therapeutic effects of the vaccine were eliminated by depletion of NK cells. Interestingly, while initial antitumor immunity was mediated by NK cells, challenge experiments in surviving vaccinated mice found that tumors were rejected through memory CD8^+^ T-cell responses, suggesting that primary destruction of the tumor by NK cells can trigger adaptive immune responses [87]. A Phase I dose escalation study of CRS-100 in patients with hepatic metastases showed similar effects on NK cells. A single injection of CRS-100 upregulated expression of the NK cell maturation marker CD38 and stimulated the production of Th1 cytokines in a dose-dependent manner [69]. Moreover, this trial was the first to demonstrate the safety and tolerability of the LADD^®^ strain in a clinical setting. It appears that the development of CRS-100 has been halted in favor of constructs that incorporate TAAs which may directly stimulate antigen-specific CD8^+^ T-cell responses [17].

### 5.5. CRS-207

CRS-207 is an Lm vaccine expressing the tumor-associated antigen mesothelin as a fusion protein with a modified form of ActA [69]. Mesothelin is a glycoprotein that is normally confined to serosal cells lining the pleura, peritoneum, and pericardium [88]. Overexpression of mesothelin has been observed in pancreatic, ovarian, lung, and multiple other cancers, making it an attractive target for immunotherapy [88]. Clinical trials testing CRS-207 have primarily been performed in patients with metastatic pancreatic ductal adenocarcinoma (PDAC). After Phase I testing demonstrated the safety of CRS-207 in patients with pancreatic, mesothelioma, lung, and ovarian cancers (ClinicalTrials.gov identifier NCT00585845), multiple Phase II trials in PDAC were initiated using a prime-boost strategy. In these trials, CRS-207 is given as a boost after initial priming with GVAX and cyclophosphamide (Cy/GVAX) therapy. GVAX is a cancer vaccine composed of irradiated pancreatic cancer cells engineered to secrete GM-CSF [89]. Cyclophosphamide is typically given prior to GVAX based on studies demonstrating inhibitory effects on regulatory T cells and an increased survival benefit compared to GVAX alone [89]. The use of CRS-207 in combination with Cy/GVAX has been supported by at least two observations. First, early Phase I testing of CRS-207 found an increased survival benefit in a small cohort of PDAC patients who received prior GVAX therapy compared to those who did not [69]. Moreover, preclinical studies in mouse PDAC models utilizing GVAX prime with CRS-207 boost demonstrated synergistic induction of mesothelin-specific T cells and inhibition of tumor growth [71]. Early Phase II testing in patients with metastatic PDAC demonstrated increased median OS in patients receiving GVAX with CRS-207 compared to Cy/GVAX alone (6.1 vs. 3.9 months) [71]. The induction of mesothelin-specific T cells was found to correlate with increased overall survival [71]. Interestingly, there was no observed difference in the number of circulating mesothelin-specific CD8^+^ T cells between Cy/GVAX and Cy/GVAX + CRS-207. Based on the results from this study, a Phase IIb study (ECLIPSE) was initiated which compared CRS-207 vs. Cy/GVAX with CRS-207 vs. conventional chemotherapy. Data presented at a conference hosted by the American Society for Clinical Oncology indicated no survival benefit from either CRS-207 alone (5.4 months), or the combination of CRS-207 with Cy/GVAX (3.8 months), compared to standard-of-care chemotherapy (4.6 months) [70]. Additional trials exploring CRS-207 and Cy/GVAX in combination with αPD-1 and αCTLA-4 immune checkpoint inhibitors as well as with indoleamine dioxygenase (IDO) inhibitors are underway.

These are complemented by other trials using CRS-207 in patients with mesothelioma, gynecologic, and gastroesophageal cancers, and data in mesothelioma patients have been published. These data showed that, after two infusions of CRS-207 in combination with six cycles of chemotherapy, 59% of patients exhibited a partial response, and an additional 35% experienced stable disease [72]. Further testing demonstrated an increase in tumor-infiltrating lymphocytes post-vaccination [72]. While numerous trials with CRS-207 are currently ongoing, Aduro has discontinued further clinical advancement of CRS-207.

### 5.6. ADU-623

ADU-623 is currently the only Lm vaccine being developed for the treatment of brain cancers. ADU-623 is a bivalent vaccine that expresses EGFRvIII and NY-ESO-1 antigens [73]. EGFRvIII is the most commonly occurring EGFR (epidermal growth factor receptor) mutation, producing a neoantigen formed from the spontaneous deletion of exons 2–7 which encode sequences in the extracellular domain [90]. Oncogenic EGFRvIII signaling may directly promote tumor growth and is frequently present in glioblastoma, lung, and other cancers [90]. NY-ESO is a cancer-testis antigen, the expression of which is restricted to germ cells in healthy adults. However, aberrant expression of NY-ESO-1 has been observed in neuroblastoma, esophageal, and other cancers [91]. Currently, a Phase I trial assessing ADU-623 in patients with high-grade astrocytomas is ongoing (ClinicalTrials.gov identifier NCT01967758). The trial is expected to be completed in December 2018.

### 5.7. JNJ-64041757 and JNJ-64041809

In 2014, Aduro licensed the Lm vaccines JNJ-64041757 and JNJ-64041809 to Janssen Biotech. JNJ-64041757 (previously known as ADU-214) is a bivalent Lm vaccine expressing EGFRvIII and mesothelin antigens [4]. Early reports from a Phase I trial in non-small cell lung cancer patients suggest that JNJ-64041757 exhibits a safety profile similar to CRS-207 and is capable of generating mesothelin-specific T-cell responses [74]. Based on these results, a subsequent Phase I/II trial assessing JNJ-64041757 alone or in combination with the αPD-1 inhibitor nivolumab was launched (ClinicalTrials.gov identifier NCT03371381). The trial is expected to be completed in March 2020. JNJ-64041809 (previously known as ADU-741) contains Lm expressing multiple prostate cancer-associated antigens [4]. Currently, it is undergoing Phase I clinical testing in patients with metastatic castration-resistant prostate cancer. A Phase II trial testing JNJ-64041809 in combination with the nonsteroidal antiandrogen apulatamide was initiated, but it was withdrawn prior to enrollment (ClinicalTrials.gov identifier NCT02906605).

### 5.8. pLADD and ADXS-NEO

During tumorigenesis, cancer cells undergo genetic alterations which can lead to the cumulative production of mutated protein sequences not found in healthy tissues [92]. These mutated proteins, termed neoantigens, may undergo proteasomal degradation and MHC loading, forming unique epitopes presented only by the tumor cells harboring the underlying mutations. In theory, vaccines targeting neoantigens may generate stronger T-cell responses due to the absence of tolerance mechanisms as well as fewer off-target effects [92]. Based on this rationale, two Lm vaccines targeting personalized neoantigens, ADXS-NEO and pLADD, were recently approved for Phase I testing. Proof of concept for ADXS-NEO was demonstrated using CT26 and MC38 colorectal cancer cell lines, and this was presented at the American Association for Cancer Research annual meeting [93,94]. The development of personalized vaccines involved whole-exome sequencing to identify tumor-specific mutations followed by analysis with predictive algorithms to identify potential neoepitopes. After screening to verify immunogenicity, the neoantigens were cloned into Lm and found to induce tumor regression upon immunization [93,94]. A Phase I trial testing ADXS-NEO in patients with metastatic head and neck, colon, and lung cancer is underway (ClinicalTrials.gov identifier NCT03265080). The personalized Lm vaccine pLADD is similarly undergoing Phase I testing in patients with microsatellite stable metastatic colorectal cancer (ClinicalTrials.gov identifier NCT03189030). In addition to personalized vaccines, Advaxis is currently developing ADXS-HOT. Because sequencing for neoantigens is a time-consuming and expensive process, ADXS-HOT is designed to be an “off-the-shelf” therapy containing a collection of frequently-mutated somatic, cancer testis, and oncofetal antigens [95]. However, the clinical details concerning this vaccine have not been released.

### 5.9. Adverse Effects of Listeria Vaccine Therapy

In general, the most common side effects of Lm vaccines are grade 1 or 2 adverse events, including transient fever, chills, nausea, vomiting, and hypotension [66,67,69,71]. Increases in circulating liver enzymes, as well as lymphopenia, have also been reported upon administration of vaccines [65,69,71]. In at least two cases, systemic listeriosis was reported after administration but was adequately treated with antibiotics [42,96]. Overall, multiple clinical trials have demonstrated an excellent safety profile with attenuated Lm vaccines in humans.

## 6. Conclusions and Future Directions

While preclinical models have shown impressive therapeutic benefits of Lm vaccines, currently, no Lm vaccines are FDA-approved. It is important to note that Lm vaccines are still relatively nascent, with most clinical trials only being initiated in recent years. As these trials advance, further improvements to Lm vaccine technology will likely be made in the coming years. Indeed, after initial clinical trials used Lm expressing a single tumor antigen, Lm vaccine technology has evolved to include vaccines expressing multiple tumor antigens. By targeting multiple antigens, it may be possible to induce therapeutic responses that are less susceptible to antigen escape. Yet, even vaccination against multiple tumor antigens may still not be sufficient to induce optimal therapeutic responses. Tumors consist of many cell types and the tumor microenvironment has been hypothesized to be a protective niche that may limit antitumor responses. Thus, many strategies moving forward are likely to combine Lm vaccines with approaches that remodel the tumor microenvironment. One such strategy has been to directly target angiogenesis using Lm vaccines. Indeed, Lm vaccines targeting tumor-associated angiogenic proteins, including CD105 and VEGFR2, have demonstrated inhibited tumor growth [97,98,99,100] as well as the ability to induce secondary antitumor immune responses via epitope spreading [98,99]. While exciting, these vaccines have not yet been tested clinically. Other studies are likely to explore combination approaches using Lm vaccines with radiation, chemotherapy, immune checkpoint inhibitors, and other drugs. These studies are expected to firmly establish the utility of Lm vaccines in the emerging immunotherapeutic landscape for the benefit of patients with a variety of malignancies.

## Figures and Tables

**Table 1:** *Listeria* cancer vaccines in clinical trials.

Vaccine	Antigen	Cancer Indication	Drug Combination	Phase	Completion	Studies	Identifier	Company
ADXS11-001	HPV 16 E7	Cervical	Vaccine alone	I	2009	[65]	N/A	Advaxis
				I/II	12/2018 ^1^		NCT02164461	
				II	04/2016		NCT01116245	
				II	10/2018 ^1^		NCT01266460	
				III	06/2021		NCT02853604	
			Vaccine + chemotherapy	II	2017	[66]	CTRI/2010/091/001232	
		Cervical and Oropharyngeal	Vaccine + αPD-1	I/II	12/2019		NCT02291055	
		Oropharyngeal	Vaccine alone	I	11/2014		NCT01598792	
				II	08/2019		NCT02002182	
		Anal	Vaccine + chemoradiation	I/II	02/2018	[67]	NCT01671488	
			Vaccine alone	II	03/2022		NCT02399813	
		Lung	Vaccine + chemotherapy	II	03/2019 ^1^		NCT02531854	
ADXS31-142	PSA	Prostate	Vaccine + αPD-1	I/II	12/2019	[68]	NCT02325557	
ADXS31-164	HER2	HER2 + Solid Tumors	Vaccine alone	I/II	12/2018		NCT02386501	
ADXS-NEO	Personal Neo-antigens	Colon, Lung, Head and Neck	Vaccine alone	I	09/2020		NCT03265080	
CRS-100 (ANZ-100)	None	Hepatic metastases	Vaccine alone	I	02/2008	[69]	NCT00327652	Aduro
CRS-207	Mesothelin	Pancreatic, Lung, Ovarian and Mesothelioma	Vaccine alone	I	02/2009	[69]	NCT00585845	
		Pancreatic	Vaccine + Cy/GVAX	II	08/2016	[70]	NCT02004262	
			Vaccine + Cy/GVAX	II	02/2017	[71]	NCT01417000	
			Vaccine + αPD-1 + Cy/GVAX	II	01/2019		NCT02243371	
			Vaccine + αPD-1 + αCTLA-4 + Cy/GVAX	II	10/2019		NCT03190265	
			Vaccine + αPD-1 + IDO1 inhibitor + Cy/GVAX	II	06/2023		NCT03006302	
		Ovarian, Fallopian and Peritoneal	Vaccine + αPD-1 + IDO1 inhibitor	I/II	12/2018		NCT02575807	
		Gastroesophageal	Vaccine + αPD-1	II	05/2019		NCT03122548	
		Mesothelioma	Vaccine + chemotherapy	I	12/2018	[72]	NCT01675765	
			Vaccine + αPD-1	II	03/2019 ^1^		NCT03175172	
ADU-623	EGFRvIII and NY-ESO-1	Brain	Vaccine alone	I	12/2018	[73]	NCT01967758	
pLADD	Personal Neo-antigens	Colorectal	Vaccine alone	I	12/2020		NCT03189030	
JNJ-64041757(ADU-214)	EGFRvIII and mesothelin	Lung	Vaccine alone	I	03/2020	[74]	NCT02592967	Janssen ^2^
			Vaccine + αPD-1	I/II	03/2022		NCT03371381	
JNJ-64041809(ADU-741)	Multiple prostate antigens	Prostate	Vaccine alone	I	06/2018		NCT02625857	
			Vaccine + anti-androgen	II	09/2018		NCT02906605	

^1^ Primary completion; ^2^ Licensed from Aduro.
